# Prevalence and associated factors of refractive error among adults in South Ethiopia, a community-based cross-sectional study

**DOI:** 10.1371/journal.pone.0298960

**Published:** 2024-03-25

**Authors:** Marshet Gete Abebe, Abiy Maru Alemayehu, Minychil Bantihun Munaw, Mikias Mered Tilahun, Henok Biruk Alemayehu

**Affiliations:** 1 Department of Ophthalmology and Optometry, Hawassa University, Comprehensive Specialized Hospital, Hawassa, Ethiopia; 2 Department of Optometry, School of Medicine, University of Gondar, Comprehensive Specialized Hospital, Gondar, Ethiopia; Chiemsee Augen Tagesklinik, Technical University of Munich, GERMANY

## Abstract

**Introduction:**

The increasing prevalence of refractive error has become a serious health issue that needs serious attention. However, there are few studies regarding the prevalence and associated factors of refractive error at the community level in Ethiopia as well as in the study area. Therefore, providing updated data is crucial to reduce the burdens of refractive error in the community.

**Objective:**

To assess the prevalence and associated factors of refractive error among adults in Hawassa City, South Ethiopia, 2023.

**Method:**

A community-based cross-sectional study was conducted on 951 adults using a multistage sampling technique from May 8 to June 8, 2023, in Hawassa City, South Ethiopia. A pretested, structured questionnaire combined with an ocular examination and a refraction procedure was used to collect data. The collected data from the Kobo Toolbox was exported to a statistical package for social sciences for analysis. Binary and multivariable logistic regression analyses were performed. A P-value of less than 0.05 was considered statistically significant in the multivariable analysis.

**Result:**

A total of 894 study participants were involved in this study with a 94.1% response rate. The prevalence of refractive error was 12.3% (95% CI: 10.2, 14.5%). Regular use of electronic devices (adjusted odds ratio = 3.64, 95% CI: 2.25, 5.91), being diabetic (adjusted odds ratio = 4.02, 95% CI: 2.16, 7.48), positive family history of refractive error (adjusted odds ratio = 2.71, 95% CI 1.59, 4.61) and positive history of cataract surgery (adjusted odds ratio = 5.17, 95% CI 2.19, 12.4) were significantly associated with refractive error.

**Conclusion and recommendation:**

The overall magnitude of refractive error in our study area was high. Regular use of electronic devices, being diabetic, positive family history of refractive error, and a positive history of cataract surgery were associated with refractive error.

## Introduction

Refractive error (RE) is a condition where the optical system of the eye fails to focus parallel rays of light on the retina. The RE occurs when there is an imbalance between the axial length and the refractive power of the eye [1]. Symptoms of RE include blurring of vision, headaches, eyestrain, and problems with focusing and seeing details at any distance. Globally, the prevalence of RE was 12% [2]. The prevalence of RE ranges from 6% to 72% in developed countries [3, 4]. In Sub-Saharan Africa, the prevalence of RE was approximately 46% [5, 6]. Hospital-based studies conducted in Gondar, Borumeda, and Arba Minch, Ethiopia showed that the prevalence of RE was 76.3%, 18.3%, and 27.5% respectively [7–9].

Globally, 2.2 billion people suffer from visual impairment (VI), and RE accounts for 88.4 million cases [10]. RE is the most common cause of visual impairment worldwide. Around 50% of the world’s vision impairment and blindness caused by RE are found in Asia [11]. According to Ethiopian national surveys, RE accounts for 33.4% of low vision and is the second leading cause of VI after cataracts [12]. RE can undermine individual performance, reduce social participation, and reduce employability. RE can also increase the economic burden on the country. Approximately US$202 billion is attributed to VI due to uncorrected RE [13]. Those above conditions result in a reduced quality of life for individuals with RE [11]. Among the top 20 causes of disability-adjusted life years, RE is one of the four non-fatal disorders [14].

Some of the factors, such as age, educational level, history of cataract surgery, family history of RE, and history of diabetes mellitus were associated with the development of RE, as reported by studies [15, 16]. Although RE cannot be completely prevented, it can be treated easily. RE can be treated with spectacle, contact lens, or refractive surgery [17].

To address the issue, multi-tiered points of delivery for refractive care services and optical dispensing units were established, together with highly qualified optometry personnel [18]. Ethiopia launched the Vision 2020 global initiative to develop a comprehensive and sustainable eye care system that will eliminate the major causes of avoidable blindness [19].

The increasing prevalence of RE in both developed and developing nations remains an urgent public health problem that needs serious attention [10, 11]. Although RE is prevalent across the world, there is limited evidence on the burden and predictors of RE among adults at the community level in Ethiopia. Hence, conducting the prevalence and associated factors gives updated information that contributes to reducing the burden of RE. In addition, this study can be used as baseline information for policymakers, the Ministry of Health, and other researchers to allocate resources for eye care service delivery.

## Method and materials

### Study design

A community-based cross-sectional study was conducted.

### Study area and period

The study was conducted in Hawassa City, South Ethiopia from May 8, 2023, to June 8, 2023. Hawassa is the capital city of the Southern Nations, Nationalities, and Peoples Region as well as the Sidama Regional State. It is located 273 kilometers (170 miles) south of Addis Ababa. According to the Ethiopian National Housing and Census Statistical Agency, the population of Hawassa city administration is expected to be 403,025 people, and out of this, 266,331 people live in the urban with an estimated household of 63,412 [20]. There are 20 kebeles (The smallest administrative unit of Ethiopia, contained within a woreda) in the city. Five government health centers and four hospitals are found in Hawassa City. In general, there are four private eye clinics and one comprehensive, specialized hospital that provides a comprehensive eye care service that serves more than 16 million people in the catchment area. In addition, there is one general hospital that provides eye care services.

### Source and study population

All adults who lived in Hawassa City were the source population and all adults aged ≥18 years who lived for at least 6 months in households of selected kebeles in Hawassa City were the study population.

### Inclusion and exclusion criteria

All adults aged ≥18 years who lived for at least 6 months in households of selected kebeles in Hawassa city were included in the study and adults aged ≥18 years with ocular comorbidities (like corneal opacity, and active eye infection) that obscure retinoscopy reflex during the refraction, adults aged ≥18 years with an absolute blind eye, adults aged ≥18 years who were unable to respond due to serious illness, and mental illness were excluded from the study.

### Sample size and sampling procedure

#### Sample size determination

A single population proportion formula was used by considering the following assumptions:

$$n=\frac{{\left(Z_{\alpha /2}\right)}^{2}P(1-P)}{d^{2}}$$

Where;

n = sample size

Z = Value of z statistic at 95% confidence interval = 1.96

α (level of significance) = 5%

P = proportion of RE from a study in Eritrea 6.4% [21]

d = allowable maximum margin of error 2%

$$Sample\;size=\frac{3.84\times 0.064\times 0.936}{0.02^{2}}=576$$

Design effect = 1.5 and Non response rate = 10%

The final sample size was 951

#### Sampling technique and procedure

**Fig 1:**
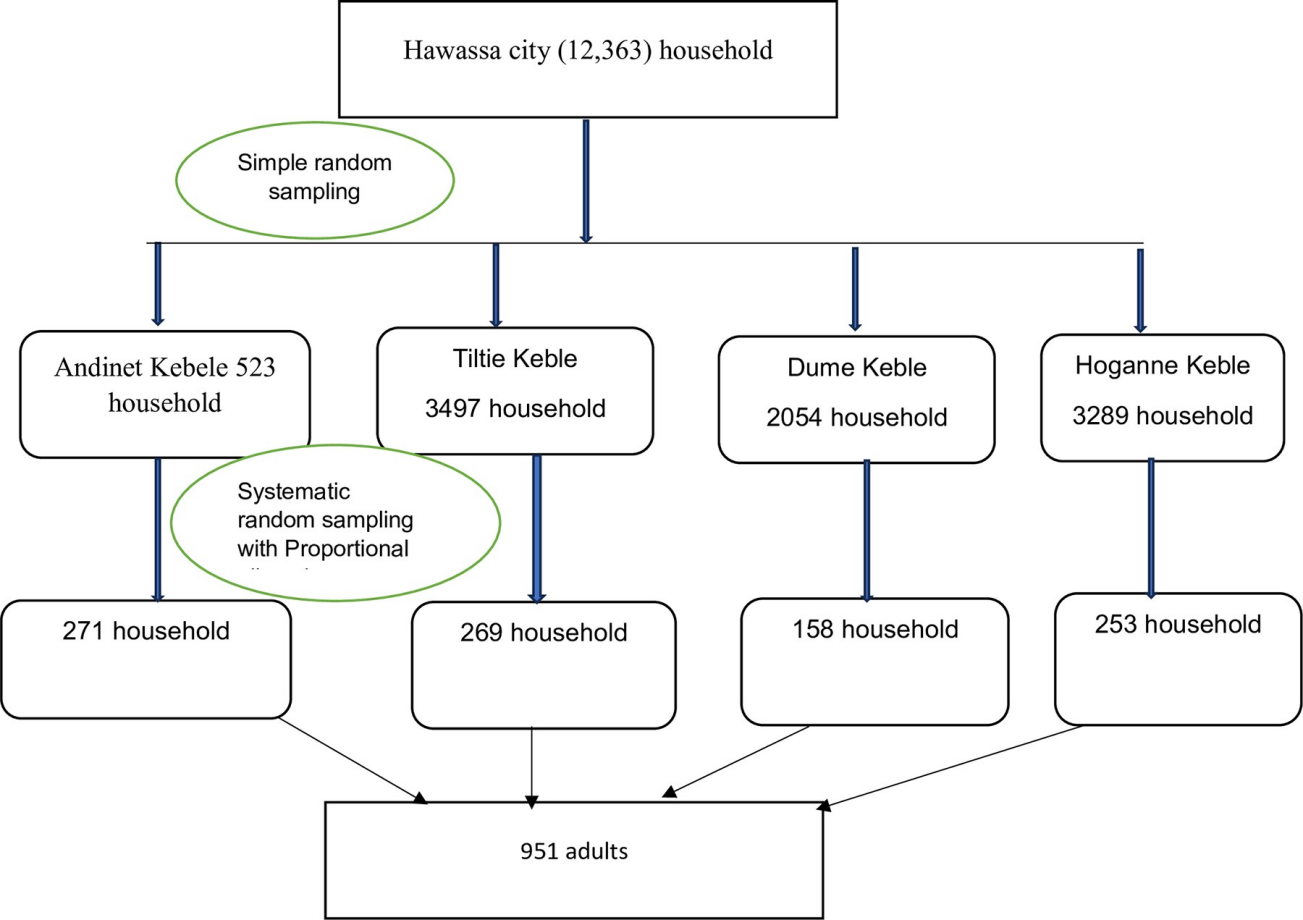
Schematic presentation of sampling technique and procedures for prevalence and associated factors of refractive error among adults in Hawassa City, South Ethiopia, 2023.

In Hawassa city, there are 20 kebeles. A multistage sampling technique was employed to select a representative sample from the city. The list of the total of kebeles was obtained from the Hawassa city administration. The four kebeles were chosen by lottery using simple random sampling. The selected four kebeles contained 12,363 of the city’s total households (63,412). The appropriate household was then picked by systematic random sampling with a K interval after the sample size was proportionally assigned based on the household size of each selected kebele Fig 1. The K interval was calculated by dividing the number of total households in the selected kebele by the total sample size (i.e., 12,363 / 951; K = 13).

Then, at random, we chose a number between 1 and 13 to choose the first family to be included in the sample, and every 13th household was included after that. For families with more than one person eligible for the study, a lottery approach was used to choose study participants. When the eligible individual was not present at the time of data collection, the residence was revisited twice. When there were no eligible persons who met the inclusion criteria in the selected household, a household listed immediately was evaluated.

### Operational definitions

RE was defined as a spherical equivalent of > +0.50 or < -0.50 diopter in either eye on subjective refraction. Myopia was defined as a spherical equivalent of < -0.50 D. High myopia was defined as a spherical equivalent of > -6.00 D [22]. Hyperopia was defined as a spherical equivalent of > +0.50 D. Astigmatism was defined as cylinder power > 0.50 D, without taking the direction of the axis into account [23]. Smoking was defined as those who smoked one stick of cigarette within the last month [24]. Sleeping Duration was defined as a longer duration when an individual sleeps for 6 hours or more and **a** short duration when an individual sleeps for less than 6 hours [25]. History of cataract surgery was defined as the examiner, facing the patient, shining the light source on the patient’s eye to see Purkinje’s reflexes like small shining bubbles. Regular use of electronic devices was defined as using mobile phones or computers, and other electronic devices at least once a day for at least two hours [26]. Family history of RE was defined as a family member (mother, father, brother, and sister) of RE diagnosed by professionals or any spectacle use [27]. History of diabetes mellitus and hypertension was defined if the individual has/had a diagnosed diabetic mellitus/ hypertension or undergoing anti-diabetes mellitus/antihypertensive treatment [28].

### Data collection tools, procedures, and quality control

#### Data collection tools, procedures

In this study, data were collected in three sections which were face-to-face interviews, ocular examinations, and refraction procedures. The data were collected by five qualified and well-trained Optometrists. A brief explanation of the purpose of the study was provided then verbal informed consent was obtained before collecting the information. An electronic data collection tool called Kobo Toolbox version 2022.4.4 was used to collect the data. A pre-tested and semi-structured interviewer-administered questionnaire adapted from previous studies [9, 29, 30] was used to conduct the data collection. The questionnaires consist of several questions to assess socio-demographic characteristics, behavioral factors, systemic co-morbidity, and clinical factors (S1 File). One supervisor (MSc in Clinical Optometry) from Hawassa University supervises the data collector every day during the data collection time.

#### Ophthalmic examination

Following the interview, all study participants received an ophthalmic examination and refraction. Optometrists performed ophthalmic examinations, which began with a VA test. Monocular and binocular unaided VA, and VA after refractive correction were measured using reduced Snellen acuity charts measured at 3 meters under normal illumination. When participants could not see a letter at 3 meters their VA was tested by reducing the testing distance and when the participant could not see letters at 1 meter, VA was determined by counting fingers, hand motion, light perception, and no light perception. Following the recording of the VA, a torch was used to inspect for the presence of any corneal opacity, cataracts, or pseudophakia/aphakia.

Finally, the optometrist set up a semi-dark room within the participant’s home for the static retinoscopy technique and retinoscopy was performed for each study participant. Objective refraction was performed using streak retinoscopy. The objective retinoscopy result was then refined using monocular subjective refraction. Subjective refraction was then recorded for each eye. Finally, the spherical equivalent was calculated for the result of subjective refraction. Study participants with a spherical equivalent of > +0.50 or < -0.50 diopter in either eye were categorized as having RE. Finally, for individuals with refractive problems, a spectacle prescription was supplied to the participant.

### Data quality control

To ensure the consistency of the data, the questionnaire was translated from English to Amharic and back again. A pre-tested Amharic version of semi-structured questions was used to ensure the reliability of the questionnaires. Before collecting data, a pretest of 48 participants (5% of the sample size) was conducted in Yirgalem, Sidama, to ensure that the questionnaire was clear, acceptable, and understandable.

To increase the quality of the data, the data collectors and one supervisor received one day of training before the actual data collection day. Training on how to utilize the Kobo Toolbox, examination procedures, and interviewing techniques was given. The supervisor closely monitored the data collection activities in the field and ensured that the collected data was complete and consistent.

### Data processing and analysis

The data collected in the Kobo Toolbox was checked for completeness and consistency. The data were exported to Microsoft Excel, cleaned, and coded with SPSS 26, and then further analysis was conducted by using SPSS. Descriptive statistics like percentage and frequency were used to summarize demographic data and categorical variables. A binary logistic regression was used to identify factors related to RE. In the bivariable analysis, variables having a P-value of less than 0.2 were entered on the multivariable logistic regression (S2 File).

The variance inflation factor (VIF) and tolerance test have been used to determine whether the independent variables were multi-collinear, and a value less than 1.05 with a tolerance less than 0.955 was found. The model’s fitness was evaluated using the Hosmer and Lemeshow goodness of fits, and the P-value was 0.76. To demonstrate the relationship between the independent and dependent variables, an adjusted odds ratio with a 95% confidence interval was computed. A P-value of less than 0.05 was considered statistically significant.

## Result

### Socio-demographic characteristics of study participants

A total of 894 participants were involved in the study, the remaining 57 individuals were non-respondents making a response rate 94.1%. 3 cases with corneal opacity and 2 cases with infection were excluded during the study. The median age of the participant was 37 years, with an interquartile range (IQR) (28–50). Out of 894 study participants, 466 (52.1%) were male, (23.0%) were private employees and 478(53.5%) had college/university educational status (Table 1).

**Table 1:** Socio-demographic characteristics of study participants among adults in Hawassa City, South Ethiopia, 2023 (n = 894).

Variables	Categories	Frequency (N)	Percent (%)
Age (year)	18–28 29–37 38–50 51–80	238 197 242 217	26.6 22.0 27.1 24.3
Sex	Male Female	466 428	52.1 47.9
Educational status	Unable to read and write Read and write Primary school Secondary school College/ University	15 63 71 267 478	1.7 7.0 7.9 29.9 53.5
Occupational status	Unemployed Farmer Housewife Student Merchant Government employee Private employee	106 22 128 93 140 199 206	11.9 2.4 14.3 10.4 15.7 22.3 23.0

n = sample size

### Systemic comorbidities, clinical and behavioral characteristics of study participants

This study reported that 69(7.7%), 58(6.5%), and 124(13.9%) of the study participants had a history of diabetic mellitus, hypertension, and a family history of RE respectively. Besides, regular use of electronic devices was found among 201(22.5%) of the study participants (Table 2).

**Table 2:** Systemic comorbidities, clinical and behavioral characteristics of study participants among adults in Hawassa City, South Ethiopia, 2023 (n = 894).

Variables	Categories	Frequency(N)	Percent (%)
Diabetes mellitus	Yes No	69 825	7.7 92.3
Hypertension	Yes No	58 836	6.5 93.5
Eye examination	Yes No	380 514	42.5 57.5
Duration of eye examination(year) (n = 380)	>3 ≤ 3	27 353	7.1 92.9
Mode of an eye examination (n = 380)	Home Traditional medicine Hospital/clinic	2 3 375	0.5 0.8 98.7
Family history of RE	Yes No	124 770	13.9 86.1
History of wearing spectacle	Yes No	24 870	2.7 97.3
Having cataract	Yes No	85 809	9.5 90.5
History of cataract surgery	Yes No	30 864	3.4 96.6
Smoking	Smoker Non-Smoker	32 862	3.6 96.4
Sleeping duration (hour)	Longer duration shorter duration	608 286	68.0 32.0
Regular use of electronic devices	Yes No	201 693	22.5 77.5

### Prevalence of RE

**Fig 2:**
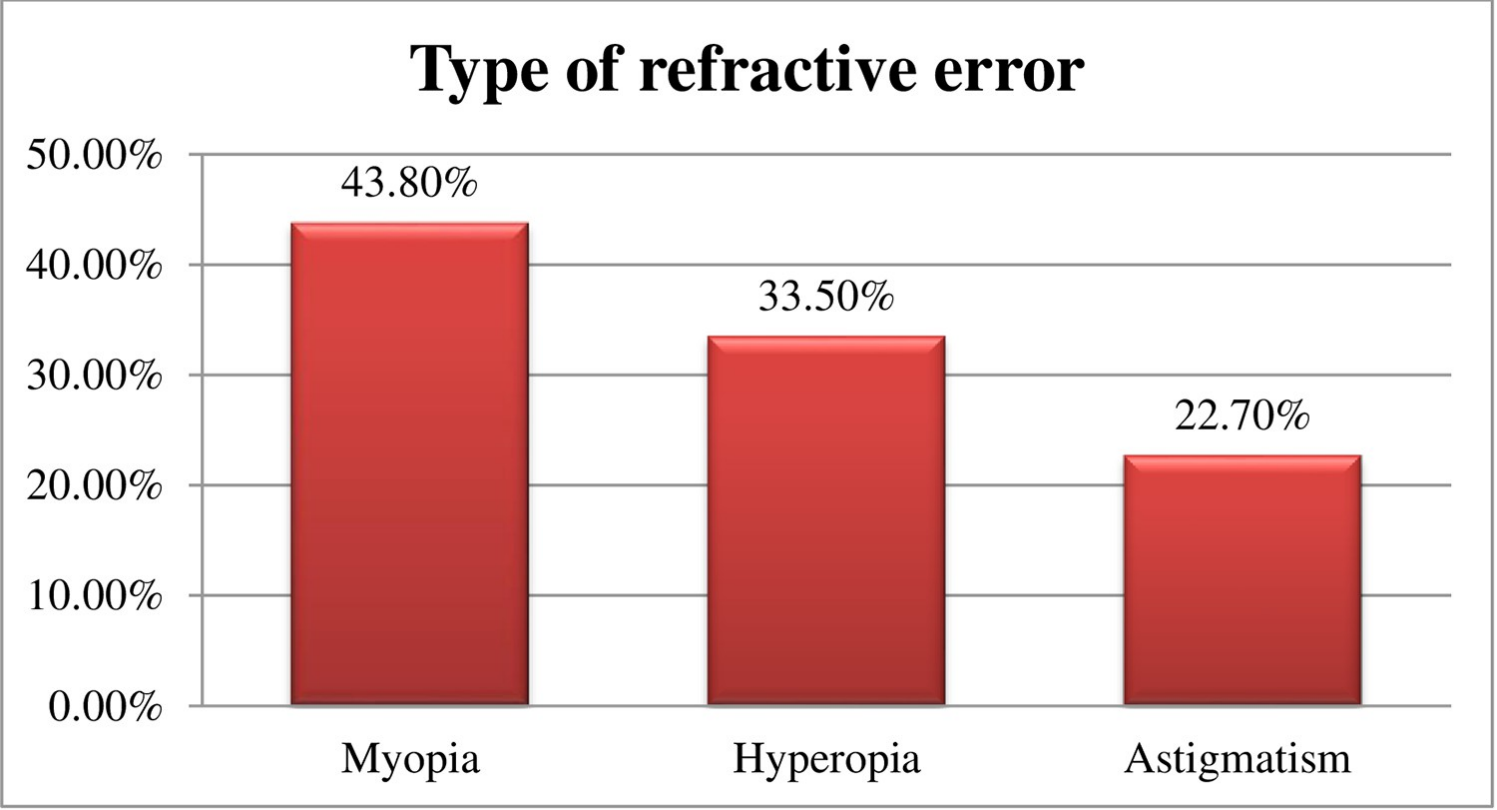
Types of refractive error among adults in Hawassa City, South Ethiopia, 2023 (n = 110).

Among the total of 894 participants, 110 (12.3%) [95% CI: 10.2, 14.5%] had a RE. The prevalence of uncorrected RE was 11.1%. This study revealed that from the total RE 43.8% of them had myopia and 2.7% had high myopia (Fig 2).

### Factors associated with RE

Bivariable and multivariable binary logistic regression was performed to identify the associated factors with RE. In bivariable binary logistic regression analysis, older age, being male, regular use of electronic devices, longer sleeping duration, positive history of diabetes mellitus, family history of RE, having cataract, and history of cataract surgery were associated with RE.

Those variables in the bivariable analysis that had a P-value less than 0.2 were entered into a multivariable binary logistic regression. A family history of RE, regular use of electronic devices, a positive history of diabetes mellitus, and a history of cataract surgery were associated with RE in multivariable logistic regression with a P-value of less than 0.05.

The odds of having RE among participants aged 51–80 years were two times more likely compared with participants aged 18–28 years (AOR = 2.08, 95% CI: 1.01–4.31).

Regular use of electronic devices was also significantly associated with RE. The odds of having RE among participants with regular use of electronic devices were 3.64 times higher compared to participants who had no regular use of electronic devices (AOR = 3.64, 95% CI: 2.25–5.91).

The odds of having RE among participants who had a positive history of diabetes mellitus were 4.02 times higher than those who had no history of diabetes mellitus (AOR = 4.02, 95% CI: 2.16–7.48).

The odds of having RE among Participants who had a family history of RE were 2.71 times more likely than participants who had no family history of RE (AOR = 2.71, 95% CI: 1.59–4.61). The odds of having RE among participants who had a history of cataract surgery were 5.17 times higher compared to participants who had no history of cataract surgery (AOR = 5.17, 95% CI: 2.19–12.4) (Table 3).

**Table 3:** Bivariable and multivariable binary logistic regression analysis for factors associated with RE among adults in Hawassa City, South Ethiopia, 2023 (n = 894).

Variable	RE		COR (95%CI)	AOR (95%CI)	P-value
	Yes	NO			
Age (year) 50–80 38–50 29–37 18–28	41 26 23 20	176 216 174 218	2.53(1.43–4.49) 1.31(0.71–2.42) 1.44(0.76–2.70) 1.00	2.08(1.01–4.31) 1.51(0.76–2.99) 1.78(0.89–3.57) 1.00	0.047 0.237 0.100
Sex Male Female	64 46	402 382	1.32(0.88–1.98) 1.00	1.18(0.75–1.85) 1.00	0.457
Regular use of electronic devices (hours) Yes No	51 59	150 634	3.65(2.41–5.53) 1.00	3.64(2.25–5.91) 1.00	< 0.001
Sleeping duration (hour) Longer Shorter	85 25	523 261	1.69 (1.06–2.71) 1.00	1.38(0.83–2.30) 1.00	0.208
Diabetes mellitus Yes No	30 80	39 745	7.16 (4.2–12.1) 1.00	4.02(2.16–7.48) 1.00	< 0.001
Family history RE Yes No	31 79	93 691	2.91 (1.82–4.65) 1.00	2.71(1.59–4.61) 1.00	< 0.001
Having cataract Yes No	17 93	68 716	1.92 (1.08–3.41) 1.00	1.60(0.79–3.25) 1.00	0.187
History of cataract surgery Yes No	16 94	14 770	9.36 (4.42–19.79) 1.00	5.17(2.19–12.4) 1.00	< 0.001

COR: crude odds ratio  AOR: adjusted odds ratio

## Discussion

The prevalence and associated factors of RE were assessed in this community-based cross-sectional study among adults in Hawssa City, South Ethiopia.

The finding of this study revealed that the prevalence of RE was 12.3% (95% CI: 10.2–14.5%). This result was in line with the study conducted in Bogota, Colombia 12.5% [29]. Both studies used similar study designs, which may account for this similarity.

On the other hand, the finding of this study was lower than studies conducted in Gondar Northwest Ethiopia 35.6% [31], Borumed Ethiopia 18.3% [7], and London United Kingdom 54% [32]. In this case, the discrepancy may be due to the socio-demographic characteristics of the study population and the study setting. As an example, the study done in Gondar was conducted among pregnant women. During pregnancy, corneal curvature and central corneal thickness increase substantially, while intraocular pressure decreases. Those physiological changes contribute to RE, which may lead to an increase in the prevalence of RE [33]. Furthermore, the study in Borumed, Ethiopia, was hospital-based. Given that most patients go to the hospital for vision difficulties, this could overestimate the magnitude of RE. Furthermore, a study in London, United Kingdom, was conducted among older persons, as age causes structural changes in the ocular system, which increase the magnitude of RE [34].

The current study’s results were greater than those obtained in Eritrea 6.4% [35], Kenya 7.4% [36], and Durban South Africa [37]. This difference may be due to variations in the method they employed and cut-off points for RE. The study in Eritrea employed a definition of RE with a VA of 6/12 or worse, which excluded participants who had RE with a VA better than 6/12, which may reduce the prevalence of RE. A study done in Durban, South Africa only included 15- to 24-year-olds, but this study included all persons 18 years and above. Several ocular diseases (diabetic retinopathy, glaucoma, and cataracts) and structural changes (retinal degeneration) in the ocular system are common among older adults and thus lead to RE. Since ocular growth stabilizes at older ages, RE risk factors will likely differ from those of younger ages due to ocular growth stability and slight changes in biometrics [34]. Because of age-related ocular disorders that increase the prevalence of RE, the above condition causes an increase in RE. Furthermore, the result of a study conducted in Bangladesh 4.7% [38] was lower than in this study; this discrepancy might be caused by the difference in the study population.

The odds of having RE among participants who had a history of diabetes mellitus were 4.02 times higher compared to participants who had no diabetes mellitus. This result is comparable with the studies conducted in Borumed, Ethiopia, and Yunnan, China [7, 39]. Clinical research has demonstrated that transient RE shifts are related to blood glucose levels. Increasing glucose may decrease the osmotic pressure of aqueous humor, leading to a flow of water from the aqueous humor into the lens, resulting in functional and morphologic changes in the lens. As a result of changes in lens refractive index, diabetics are more likely to develop RE [40, 41].

The odds of having RE among participants who underwent cataract surgery were 5.17 times higher than those participants who had no history of cataract surgery. This result was supported by the study conducted in South India [42]. Cataract surgery induces RE in different ways, which can be in preoperative (errors in biometry parameters, Pre-existing systemic & ocular comorbidities, Pre-existing uncorrected corneal astigmatism >1.00 DC), intraoperative (surgical variations of incision size, incision location, Use of sutures), or postoperative (shift in IOL position) conditions [43–45].

The odds of having RE in participants who had a family history of RE was 2.71 times higher than in participants who had no family history of RE. This result is comparable with the studies conducted in Arba Minch, Ethiopia, and East China [9, 30]. Studies have found considerable relationships between first-degree relatives’ RE. Research has shown that RE aggregates significantly within families. It has been reported that the heritability of RE ranges from 50% to 90% within various populations [46, 47].

The odds of having RE among participants who have regular use of electronic devices were 3.64 times higher than participants who have no regular use of electronic devices. This result was consistent with a study conducted in Gondar, Northwest Ethiopia, and Rohtak India [48, 49]. Staring at the computer for an extended period causes prolonged accommodation and muscle fatigue, which might result in a transient shift in the refractive status of the eye [50]. In addition, staring at the computer for an extended time will cause dry eye, which will affect the refractive power of the cornea [51].

## Strengths and limitations of the study

Both objective and subjective full refraction procedure was performed to determine the refractive status of the eye. As the study is community-based it is more representative than institution-based studies.

A cross-sectional study design does not reveal a cause-and-effect relationship between dependent and independent variables. Recall bias was another issue due to the nature of the questionnaire to assess family history of RE and smoking.

## Conclusion

As a conclusion, the prevalence of RE in this study area was 12.3%. A family history of RE, regular use of electronic devices, a positive history of diabetes mellitus, and a history of cataract surgery were significantly associated with RE. Since most of these associated factors are modifiable (regular use of electronic devices, a positive history of diabetes mellitus, and a history of cataract surgery), eye care professionals should primarily focus on the prevention of these modifiable causes. To mitigate the burden of RE, it is recommended that eye care professionals prioritize early screening of individuals with diabetes. From a perspective of minimizing post-operative RE following cataract surgery, there is a need to enhance preoperative evaluation and intraoperative care.

## Supporting information

S1 File English version of questionnaire. (DOCX)

S2 File Data used for analysis including data on refractive error and associated factors. (SAV)
