# Supplementary material for: Prevalence and associated factors of refractive error among adults in South Ethiopia, a community-based cross-sectional study
Source: PLoS One. 2024 Mar 25;19(3):e0298960. doi: 10.1371/journal.pone.0298960 (PMC10962790; doi:10.1371/journal.pone.0298960)
Supplement: S1 File — (DOCX) [file pone.0298960.s001.docx]

**General information**

Code ______________ Kebele______

Data collection date_______________________________

Name of data collector_____________________________

**Part I: Assessment of Socio-demographic Variables**

| S.No | Question | Response | Remark |
| --- | --- | --- | --- |
| 101 | Age (year) | year |  |
| 102 | Sex | 1. Male 2. Female |  |
| 103 | Educational status | 1. Unable to read and write 2. Read and write 3. Primary school 4. Secondary school   5. College/University completed and above |  |
| 104 | Occupational status? | 1. Government employee 2. Private employee 3. Housewife 4. Merchant 5. Farmer 6. unemployed   7. Student |  |

**Part II: Behavior and personal related questions**

| 201 | Do you smoke a cigarette? | 1. Yes 2. No | If no, go to question no.203 |
| --- | --- | --- | --- |
| 202 | If yes How many sticks did you smoke within the last month? | _________number |  |
| 203 | Do you use electronic devices (mobile, computer)? | 1. Yes 2. No | If no, go to question no 205 |
| 204 | For how long do you use electronic devices per day? | _______hour/day |  |
| 205 | For how long do you sleep in a day? | _______hour/night |  |

**Part III Clinical Data and systemic comorbidity**

| 301 | Do you have a DM (have you been diagnosed before/taken any medication)? | 1. Yes 2. No 3. I don’t know |  |
| --- | --- | --- | --- |
| 302 | Do you have HTN (have you been diagnosed before/taken any medication)? | 1. Yes 2. No 3. I don’t know |  |
| 303 | Is there any diagnosed refractive error (spectacle use) in your family? | 1. Yes 2. No | If no, go to question no. 305 |
| 304 | If yes, for what purpose do they use the spectacle? | 1. For distance 2. for reading |  |
| 305 | Have you ever had an eye examination/ checkup before? | 1. Yes 2. No | If no, go to question no. 308 |
| 306 | If yes where did you examine your eye? | 1. hospital/clinic  2. At home  3. Traditional medicine |  |
| 307 | If yes when was the last time you visit did you visit the eye care service center | ----------------------------year |  |
| 308 | Do you have any history of cataract surgery before? | 1. Yes 2. No |  |
| 309 | Do you have a history of wearing a spectacle? | 1. Yes 2. No |  |

**Part: VI Data on ocular examination**

|  |  | **Right eye** | **Left eye** | **Both eye** |
| --- | --- | --- | --- | --- |
| 401. | Distance presenting VA |  |  |  |
| 402 | Distance VA with current spectacle |  |  |  |
| 403. | Retinoscopy result |  |  |  |
| 404 | Subjective result |  |  |  |
| 405. | Ocular findings | Normal  Cataract  Cataract surgery 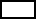 | Normal  Cataract  Cataract surgery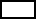 | Normal  Cataract  Cataract surgery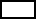 |

**Thank you very much for taking part in this study**
